# Combined use of dexmedetomidine and propofol in monitored anesthesia care: a randomized controlled study

**DOI:** 10.1186/s12871-017-0311-9

**Published:** 2017-03-01

**Authors:** Kyu Nam Kim, Hee Jong Lee, Soo Yeon Kim, Ji Yoon Kim

**Affiliations:** Department of Anesthesiology and Pain Medicine, Hanyang University Hospital, 222, Wangsimni-ro, Seongdonggu, Seoul, 133-792 Republic of Korea

**Keywords:** Combination drug therapy, Deep sedation, Dexmedetomidine, Propofol

## Abstract

**Backgroud:**

Although propofol and dexmedetomidine have been widely used for monitored anesthesia care, their adverse effects necessitate the search for better methods. Therefore, we performed this randomized controlled trial to evaluate the combined use of propofol and dexmedetomidine.

**Methods:**

Eighty-seven adult patients undergoing hand surgery under brachial plexus block were randomly allocated to receive 1.6 μg/ml of the target effect site concentration of propofol (P group) and infusion of 0.4 μg/kg/h dexmedetomidine following a loading dose of 1.0 μg/kg for 10 min (D group). The M group received a half-dose of both drugs simultaneously. The maintenance dose was adjusted to maintain an Observer Assessment of Alertness/Sedation score of 3. Cardiorespiratory variables, adverse effects, and drug efficacy were observed.

**Results:**

The significantly higher mean arterial pressure (mmHg) in the D group [P group 86.9 (12.6), D group 96.0 (12.2), M group 85.6 (10.6), *p* = 0.004)] and a significantly higher heart rate (beat/min) in the P group were observed [P group 67.3 (9.0), D group 57.8 (6.9), M group 59.2 (7.4), *p* < 0.001)]. The M group had a significant lower incidence of airway obstruction (*p* < 0.001) and the D group had a higher incidence of bradycardia requiring atropine (*p* = 0.001). The P group had higher incidences of hypoxia (*p* = 0.001), spontaneous movement (*p* < 0.001) and agitation (*p* = 0.001). The satisfaction scores of the patients (*p* = 0.007) and surgeon (*p* < 0.001) were higher in the M group. Onset time was significantly longer in the D group (*p* < 0.001).

**Conclusions:**

The combined use of propofol and dexmedetomidine provided cardiovascular stability with decreased adverse effects. Additionally, it led to a similar onset time of propofol and achieved higher satisfaction scores.

**Trial registration:**

KCT0001284. Retrospectively registered 25 November 2014.

## Background

Monitored anesthesia care (MAC) has been used to provide sedation, comfort, memory loss and relief from anxiety during therapeutic or diagnostic procedures with sedation and analgesia [1, 2]. Because respiratory depression is associated with the most serious patient injuries during MAC [3], the optimal state of MAC is the maintenance of sedation and normal cardiovascular functions without severe respiratory depression and airway obstruction. The capability to rapidly modulate the depth of sedation when necessary is also an important requirement of MAC. Several sedative, analgesics and narcotics are used to achieve these objectives while minimizing adverse effects.

Among these drugs, propofol provides antiemetic properties, high quality sedation, and rapid onset and recovery times [4, 5]. Additionally, a consistent target effect site concentration can be maintained without overdose of the drug through target controlled infusion (TCI) technology [6]. Nevertheless, propofol has some adverse effects such as severe respiratory depression and hypotension, which highlight the need to find better drugs for MAC [7, 8].

Dexmedetomidine, a highly selective α_2_-adrenergic receptor agonist, has analgesic and sedative properties without significant respiratory depression [9, 10]. Although less significant respiratory depression is prominent merits of dexmedetomidine, the adverse effects of dexmedetomidine include a dose-dependent decrease in blood pressure and heart rate due to its sympatholytic effects [11, 12]. In the absence of an ideal sedative agent, there is great interest in combining different agents to maximize efficacy and minimize adverse effects, with some studies finding that these combinations have significant benefits over single agents [13, 14]. In this study, we hypothesized that the combinatory use of propofol and dexmedetomidine would reduce adverse effects such as respiratory depression and cardiovascular depression and improve efficacy as measured by early onset and recovery time. Therefore, we performed this prospective, randomized, controlled double-blinded trial to evaluate the efficacy and safety of the combinatory use of propofol and dexmedetomidine.

## Methods

### Participant selection

After approval by the Institutional Review Board (IRB) of Hanyang University Hospital, Seoul, Korea, this study was registered at http://cris.nih.go.kr (Clinical Research Information Service, registration number: KCT0001284). Adult patients between 20 and 75 years of age who were scheduled for elective hand surgeries under brachial plexus block were included in this randomized control trial after obtaining written informed consent. Only patients who wanted sedation were included. Patients were excluded if they met the following criteria: (1) American Society of Anesthesiologists (ASA) physical status more than IV; (2) impaired cognitive function; and (3) obstructive sleep apnea, neuropsychiatric, cardiovascular, respiratory, renal or liver disorders.

Eligible patients were randomly assigned to three groups and the sequence of the sedation procedure was allocated by opening sealed envelopes before monitoring the patients. These envelopes contained a pre-determined group, which was randomly assigned using a random number generator in the Excel program by author (LHJ).

### Study groups

There are three groups in this study. In the P group, 1.6 μg/ml of initial target effect site concentration (Ce) of propofol (2% Fresofol®, Fresenius Kabi, Korea Ltd, Korea) was infused through a TCI pump (Orchestra® Base Primea, Fresenius Kabi, Brezins, France). For dose maintenance, the propofol was titrated by 0.2 μg/ml of Ce depending on the Observer Assessment of Alertness/Sedation (OAA/S) score [15]. Patients in the D group received an infusion of 0.4 μg/kg/h dexmedetomidine (Precedex®, Hospira Inc., Lake Forest, USA) following a loading dose of 1.0 μg/kg over 10 min. And then, the dose of dexmedetomidine was adjusted by 0.08 μg/kg/h according to the OAA/S score. In the M group, 0.8 μg/ml of initial Ce of propofol was infused through an Orchestra® Base Primea TCI pump and a loading dose of dexmedetomidine of 0.5 μg/kg was infused over 10 min together. Then, 0.2 μg/kg/h dexmedetomidine was infused for the maintenance dose. During the sedation, the maintenance doses of propofol and dexmedetomidine were titrated by the same proportion depending on the OAA/S score. The dose of propofol was adjusted by 0.2 μg/ml of Ce and the dose of dexmedetomidine was adjusted by 0.04 μg/kg/h simultaneously. The maintenance dose of each group was adjusted by one of the authors to maintain the OAA/S score of 3 in all study groups.

### Study protocol

Brachial plexus block was conducted using the same predefined protocol without premedication. All anesthetic procedures and surgeries were performed by the same anesthesiologist and surgical team. After arrival to the operating room, the patient’s vital signs were monitored by noninvasive blood pressure measurement, electrocardiography, and pulse oximetry. Respiratory variables such as end-tidal CO_2_ and respiratory rate were monitored by a side-stream infrared gas analyzer (Drager-VAMOS®, Drager Medical, Lubeck, Germany). Supplemental oxygen (4 L/min) was given to all patients. The axillary brachial plexus block was performed with 0.75% ropivacaine 20 ml under ultrasound-guided techniques. After adequate surgical anesthesia has been achieved, patients received a sedative in accordance with the method above. During sedation, all patients were maintained in the supine position and the sedation statuses of the patients were evaluated by the OAA/S score and bispectral index (BIS) monitoring. All unnecessary noise was minimized during sedation.

### Assessment of drug effect

The primary endpoints were the changes of mean arterial pressure and the extent of airway obstruction (1 = patent airway, 2 = airway obstruction alleviated by jaw thrust, 3 = airway obstruction relieved by positive mask ventilation). The time to achieving the target depth of sedation (OAA/S score of 3) was measured by calculating the time from injection to an OAA/S score of 3. In addition, the time to achieving BIS score of 70 was also measured. During the sedation procedure, vital signs and sedation status including OAA/S scores, BIS scores, mean arterial pressure, heart rate, SpO_2_, end-tidal CO_2_ and respiratory rate were recorded at the following times: (1) before the injection of the drug (T0); (2) 5 min after infusion (T1); (3) achieving the target mental status (OAA/S score 3) (T2); (4) 15 min after achieving the target mental status (T3); (5) 30 min after achieving the target mental status (T4); (6) termination of infusion (T5); and (7) an alert mental status (T6).

In addition to the occurrence of airway obstruction, the incidence of adverse events such as hypoxia (SpO_2_ < 90% for > 10 s), spontaneous movements, cough, nausea, vomiting, agitation, and the administration of atropine and ephedrine were also assessed. In cases with a heart rate < 45 beats/min or a more than 30% decrease in mean arterial pressure from baseline values, 0.5 mg atropine and 5 mg ephedrine were used, respectively. Spontaneous movements were recorded when movements of the upper or lower extremity occurred more than three times. Agitation was defined as non-cooperative and non-purposeful motor restlessness. The total dose of infused drug and the recovery time from the termination of injection to an OAA/S score of 5 and BIS score of 90 were also measured. To compare the dose rates of the infused drugs, we used the value of the total dose of infused drug per body weight and infusion time. After surgery, the incidence of awareness and recall during sedation was examined. The satisfaction of the patients and surgeon, blinded to group assignment, was evaluated using a visual analog scale (VAS) of 0 to 100. All data were assessed by another anesthesiologist who was blinded to group assignment.

### Justification of sample size and Statistical analysis

According to a previous study that compared the effectiveness of dexmedetomidine and propofol target-controlled infusion for sedation [16], mean arterial pressure of propofol was 94.7 mmHg with a standard deviation of 12.3 mmHg. We considered 11 mmHg to be a meaningful difference and the calculated sample size was 27 patients in each group with an assumed an α error of 5% and ß error of 10%. Accounting for a dropout rate of 5%, 30 patients were allocated to each group.

Categorical data were expressed as numbers of patients (percentages as appropriate) and compared using Pearson’s chi-square test with Fisher’s exact test. Continuous data were expressed as mean (standard deviation). After a normality test was performed using the Shapiro-Wilk test, continuous data was compared using one-way analysis of variance (ANOVA) and a post hoc test (Duncan). Other data was analyzed by the Kruskal-Wallis test with the Mann-Whitney *U*-test, and *p* values were adjusted with Bonferroni’s correction. Repeated measures ANOVA was used to compare hemodynamic and respiratory variables over time and the incidence of adverse events was analyzed using the Chi-square test with adjusted *p* values by Bonferroni’s correction. Statistical analysis was performed with SPSS software (version 21.0 SPSS Ins., Chicago, USA). *P* < 0.05 was considered statistically significant.

## Results

**Fig. 1:**
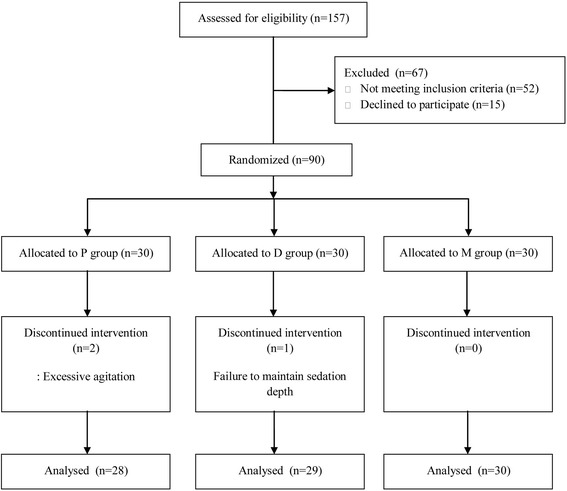
Flow diagram of patient recruitment and exclusion criteria for the study

Among 157 patients who were assessed for eligibility from August 2014 until August 2015, 67 patients were excluded because of not meeting the inclusion criteria or declining to participate. As a result, 90 patients were randomly assigned to each group by a predefined method. In P group, two patients showed so excessive agitation that general anesthesia was needed and in D group, one patient was excluded from analysis because the sedation level of the patient was deeper than the designated state per our protocol. Consequently, the data from 87 patients were analyzed in this study (Fig. 1). Patient demographic data are summarized in Table 1 and there were no differences in patient characteristics between the three groups.

**Table 1:** Patients demographic data

Variable	P group (*n* = 28)	D group (*n* = 29)	M group (*n* = 30)
Age (years)	45.5 (14.3)	47.8 (15.2)	49.6 (18.1)
Male sex	15 (53.6)	11 (37.9)	10 (33.3)
Height (cm)	166.8 (9.5)	163.8 (9.0)	164.5 (9.4)
Weight (kg)	64.0 (12.0)	61.7 (9.9)	60.3 (10.2)
Body mass index (kg/m^2^)	22.9 (3.5)	22.9 (2.6)	22.7 (2.9)
ASA physical status			
I/II	15 (53.6)/13 (46.4)	11 (37.9)/18 (62.1)	10 (33.3)/20 (66.7)
Duration of anesthesia (minute)	116.3 (30.6)	126.7 (43.1)	110.7 (33.5)
Hypertension	2 (7.1)	6 (20.7)	3 (10)

Values are numbers of patients (%), or mean (standard deviation)

### Changes in hemodynamic and respiratory variables

**Fig. 2:**
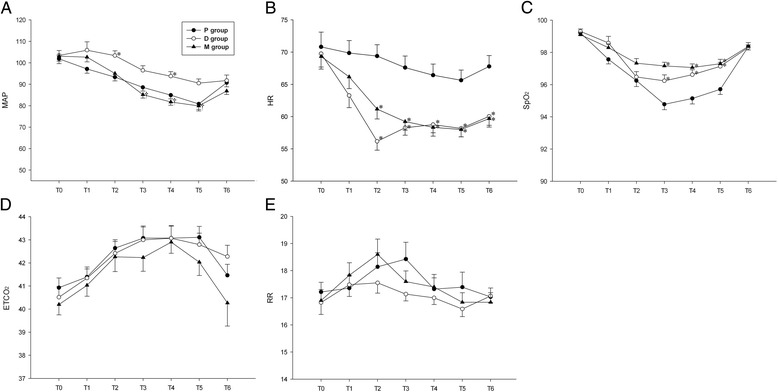
Cardiorespiratory variables during sedation. **a** Mean arterial pressure (MAP) in mmHg. **b** Heart rate (HR) in beats per minute. **c** Pulse oximetry (SpO_2_) in percentage. **d** End-tidal CO_2_ in mmHg. **e** Respiratory rate (RR) in number of respirations per minute. All data are presented as mean and standard error. *: *P* < 0.05 compared to the P group, †: *P* < 0.05 compared to the D group

The reduction of mean arterial pressure (mmHg) in the D group was significantly less than other groups [P group 86.9 (12.6), D group 96.0 (12.2), M group 85.6 (10.6), *p* = 0.004)] (Fig. 2a) and the reduction of heart rate (beat/min) in the P group was significantly less than other groups [P group 67.3 (9.0), D group 57.8 (6.9), M group 59.2 (7.4), *p* < 0.001)] (Fig. 2b). Although there were no differences in end-tidal CO_2_ (*p* = 0.56) (Fig. 2d) and respiratory rate (*p* = 0.38) (Fig. 2e) between groups, the P group had a significantly lower SpO_2_ (*p* < 0.001) (Fig. 2c).

### Adverse events

There was a significant lower incidence of the extent of airway obstruction (*p* < 0.001) in the M group (Table 2). The P group had a higher incidence of hypoxia (*p* = 0.001), spontaneous movement (*p* < 0.001) and agitation (*p* = 0.001) than other groups. The incidence of bradycardia requiring atropine was significantly greater in the D group (*p* = 0.001). No episodes of nausea, vomiting, or hypotension were found and there were no differences in the occurrence of cough (*p* = 0.16) between groups.

**Table 2:** Incidence of adverse events

Variable	P group (*n* = 28)	D group (*n* = 29)	M group (*n* = 30)	*P* value
Airway obstruction				
1/2/3	15(53.6)/13(46.4)/0(0)	21(72.4)/8(27.6)/0(0)	29(96.7)/1(3.3)/0(0)*^†^	<0.001
Hypoxia	12(42.9)	4 (13.8)*	1(3.3)*	0.001
Spontaneous movement	10 (35.7)	1 (3.4)*	0 (0)*	<0.001
Cough	5 (17.9)	3 (10.3)	1(3.3)	0.16
Nausea	0 (0)	0 (0)	0 (0)	1.00
Vomiting	0 (0)	0 (0)	0 (0)	1.00
Agitation	6 (21.4)	0 (0)*	0 (0)*	0.001
Bradycardia requiring atropine	1 (3.6)	8 (27.6)*	0 (0)^†^	0.001
Hypotension requiring ephedrine	0 (0)	0 (0)	0 (0)	1.00

Values are numbers of patients (%)

*P < 0.05 compared to the P group, ^†^ *P* < 0.05 compared to the D group

### Onset and recovery time and dose rate of drug infusion

Although there was a significantly longer time (seconds) to achieve the target depth of sedation in the D group [P group 502.7 (149.5), D group 709.1 (106.0), M group 538.9 (81.1), *p* < 0.001)], there was no difference in recovery time among the groups (*p* = 0.07) (Table 3). The dose rate of propofol infusion in the P group and of dexmedetomidine infusion in the D group were 3.54 (1.01) mg/kg/h and 1.26 (0.37) μg/kg/h, respectively. In total, 1.59 (0.56) mg/kg/h of propofol and 0.61 (0.17) μg/kg/h of dexmedetomidine were infused in the M group. In comparison with the half-dose of drug infusion in the P and D groups, there were no differences in the dose rates of drug infusion.

**Table 3:** Comparison of onset and recovery time, dose rate of drug infusion, and satisfaction scores

Variable	P group (*n* = 28)	D group (*n* = 29)	M group (*n* = 30)	*P* value
Onset time (seconds)				
Time to OAA/S score 3	502.7 (149.5)	709.1 (106.0)*	538.9 (81.1)^†^	<0.001
Time to BIS 70	590.3 (146.8)	809.3 (106.7)*	621.1 (96.0)^†^	<0.001
Recovery time (seconds)				
Time to OAA/S 5	478.8 (178.4)	580.9 (178.1)	495.5 (178.8)	0.07
Time to BIS 90	585.2 (188.4)	682.0 (179.2)	585.9 (188.6)	0.08
Dose rate of drug infusion				
Propofol (mg/kg/h)	3.54 (1.01)		1.59 (0.56)	
Dexmedetomidine (μg/kg/h)		1.26 (0.37)	0.61 (0.17)	
Patient satisfaction score (VAS)	90.0 (7.9)	89.2 (9.2)	95.0 (4.7)*^†^	0.007
Surgeon satisfaction score (VAS)	81.0 (10.4)	87.3 (8.1)*	93.7 (5.9)*^†^	<0.001

Values are mean (standard deviation)

*OAA/S score* Observer Assessment of Alertness/Sedation score, *BIS* bispectral index, *VAS* visual analog scale

*P < 0.05 compared to the P group, ^†^ *P* < 0.05 compared to the D group

### The incidence of awareness and the degree of satisfaction

There were no incidences of awareness and recall during sedation among the three groups. The higher VAS score of patient satisfaction was found in M group [P group 90.0 (7.9), D group 89.2 (9.2), M group 95.0 (4.7), *p* = 0.007] and the VAS score of surgeon satisfaction was significantly different in each group [P group 81.0 (10.4), D group 87.3 (8.1), M group 93.7 (5.9), *p* < 0.001)] (Table 3).

## Discussion

We performed this randomized, controlled, double-blinded trial to evaluate the efficacy and safety of the combinatory use of propofol and dexmedetomidine at half of their usual doses. Our study demonstrated that the combinatory use of propofol and dexmedetomidine provided cardiovascular stability, early onset time and higher satisfaction scores without delayed recovery time and adverse effects such as airway obstruction, hypoxia, and spontaneous movement.

According to our study, the reduction of mean arterial pressure in the D group was significantly less than other groups, and mean arterial pressure actually rather increased 5 min after infusion (Fig. 2a). The rapid injection of a loading dose of dexmedetomidine can have biphasic effects on blood pressure, with temporary increases in blood pressure by a direct α_2_-adrenoceptor-induced vasoconstrictive response in the peripheral vasculature followed by a lower mean arterial pressure due to decreased sympathetic outflow [9, 12]. This biphasic trend in blood pressure was observed in the D group, but temporary increases of blood pressure were not observed in the M group. In terms of heart rates, dexmedetomidine can cause bradycardia due to its well-known sympatholytic effects [12, 17, 18]. The heart rates in both the P and M groups were decreased after infusion. However, considering that the D group required the frequent use of atropine (27.6%) to maintain heart rates, while the M group did not require atropine, it is clear that the combination of propofol and dexmedetomidine helped maintain heart rates. After taking these results into consideration, we suggest that the combination of propofol and dexmedetomidine provided cardiovascular stability.

As described above, respiratory depression is the most significant adverse effect during MAC [3]. Unlike propofol, in which the incidence rate of hypoxemia was reported to be 11% [7], sedation with dexmedetomidine has a mechanism similar to natural sleep with hyperpolarization of norepinephrine receptors in the locus cereleus [19]. The locus cereleus plays an essential part in regulating sleep and the modulation of respiratory controls [20]. Therefore, the effects of dexmedetomidine on respiration and ventilation are minimal and several previous studies already have revealed the minimal changes in respiratory variables such as oxygen saturation, arterial carbon dioxide, respiratory rates and arterial pH [9, 21, 22]. Similar to the results of these studies, oxygen saturation in the D group was significantly higher than in the P group. However, as the depth of sedation increases, dexmedetomidine can cause indirect respiratory depression due to respiratory obstruction from the relaxation of the pharyngeal muscle tone [23]. This obstruction, which results in apnea, is resolved by applying slight jaw thrust. Because relatively deep sedation was maintained in our study, the incidence of airway obstruction and hypoxia was similar between the P and D groups. Despite the equivalent sedation levels of the other groups, our study revealed that the combination of propofol and dexmedetomidine resulted in a significantly lower incidence of airway obstruction and hypoxia.

In order to avoid transient hypertension, the slow injection of dexmedetomidine was required [10], which can result in slower onset of sedation. The time to OAA/S 3 or BIS 70 of the D group, which was longer than the other groups by about 3 min, also implies the delayed onset of sedation for dexmedetomidine, which correlates well with the results of a previous study [11]. The combined use of propofol and dexmedetomidine overcomes this limitation and results in an onset time similar to that of propofol. As the recovery time from sedation with dexmedetomidine and propofol are known to be equivalent [18, 24], there were no differences in recovery time between the three groups.

Our study also showed that patients and surgeons were more satisfied with the combined use of propofol and dexmedetomidine. Patients in the P group felt discomfort during the injection of propofol while those in the D group experienced a slower onset of dexmedetomidine. Surgeon satisfaction was significantly different between groups. The lowest satisfaction in the P group was due to the high incidence of agitation and spontaneous movement. Spontaneous movement caused by propofol injection, with rates of 5.5 to 22%, correlates with imbalances in excitatory inhibitory neurotransmitters [25–27]. Because spontaneous movement during procedures under MAC cancompromise patient safety, a low incidence of spontaneous movement closely related not only with safety, but also with a smoother procedure due to less frequent interruptions. Similar to patient satisfaction, the slower onset of dexmedetomidine was the reason why surgeon satisfaction scores in the D group were lower than in the M group. The high level of surgeon satisfaction in the M group was observed because of the early onset time and the absence of other complications.

Our study has several limitations. First, although one of the advantages of dexmedetomidine is its analgesic property [9–11, 28], we could not evaluate its analgesic effects because this study was conducted under brachial plexus block. Therefore, further studies are needed to evaluate the analgesic effect when dexmedetomidine is used in combination with propofol. Another limitation is that we did not use premedication, which could have an influence on sedation level. Anxiety due to the unfamiliar operating room environment and undergoing regional anesthesia could have increased baseline blood pressure, heart rate and respiratory rate. Lastly, the accuracy of end-tidal CO_2_ monitoring is also a limitation. Although we placed the airway adapter as close as possible to the patient’s airway, some degree of measurement error of end-tidal CO_2_ is inevitable in non-intubated patients.

We expected a synergistic effect between propofol and dexmedetomidine. However, judging from the requirement of half of the usual doses of propofol and dexmedetomidine to maintain the target sedation level, the combined use of propofol and dexmedetomidine seemed to have an additive effect. Further studies are needed to accurately assess whether the combined use of propofol and dexmedetomidine has an additive effect or not.

## Conclusions

We conclude that the combination of propofol and dexmedetomidine provided cardiovascular stability without transient hypertension and bradycardia. The combination of these two agents also improved patient safety by decreasing the incidence of airway obstruction, hypoxia, spontaneous movement and agitation during deep sedation. In addition, the use of propofol and dexmedetomidine had a similar onset time as that of propofol without a delayed recovery time, and achieved higher satisfaction scores than with the use of a single drug.

## Abbreviations

ANOVA: Analysis of variance
BIS: Bispectral index
Ce: Effect site concentration
MAC: Monitored anesthesia care
OAA/S score: Observer assessment of alertness/sedation score
SpO_2_: Pulse oxygen saturation
TCI: Target controlled infusion
VAS: Visual analog scale
